# Encapsulation of Sulforaphane from Cruciferous Vegetables in mPEG-PLGA Nanoparticles Enhances Cadmium’s Inhibitory Effect on HepG2 Cells

**DOI:** 10.3390/nano15080615

**Published:** 2025-04-16

**Authors:** Ren Li, Yi Zhu

**Affiliations:** College of Food Science and Nutritional Engineering, China Agricultural University, Beijing 100107, China; b20203060482@cau.edu.cn

**Keywords:** sulforaphane, anticancer, cadmium, nanoparticle

## Abstract

Sulforaphane (SFN) is a natural isothiocyanate compound with multiple bioactive effects, abundantly found in cruciferous vegetables. SFN and cadmium (Cd) were limited in their application as chemotherapeutic agents due to insufficient cellular uptake, low bioavailability, and high systemic toxicity, respectively. In this study, mPEG-PLGA nanoparticles were used as a carrier to load Cd-γ-PGA conjugates and SFN, enabling favorable drug release under acidic microenvironments with excellent pH responsiveness. The NP-Cd-SFN nanoparticles exhibited a particle size of 102.1 nm, a zeta potential of -14.48 mV, and a PDI value of 0.257. These characteristics contribute to the nanoparticles’ prolonged circulation in the bloodstream and their ability to passively target tumors. Compared to the single-dose groups and the combined Cd + SFN group, the NP-Cd-SFN group significantly reduced the viability of HepG2 cells and increased their apoptosis rate by inducing mitochondrial oxidative stress and promoting cell apoptosis. Overall, the addition of SFN and the encapsulation of mPEG-PLGA enhanced the therapeutic effects of Cd on HepG2 cells.

## 1. Introduction

Hepatocellular carcinoma (HCC) is one of the leading causes of death from malignant tumors worldwide and poses a significant challenge to global public health [1]. Although current treatments, including chemotherapy, radiotherapy, and immunotherapy, have achieved certain therapeutic effects, there remains an urgent need for more effective and less toxic therapeutic strategies [2,3]. The combination therapy of chemotherapeutic drugs, through synergistic effects, a multi-targeted approach, and personalized treatment, significantly enhances the efficacy and safety of cancer therapy while minimizing drug resistance and side effects, making it a crucial strategy in modern cancer treatment [4,5]. The nanotherapeutic delivery systems utilize nanomaterials as drug carriers, assisting in enhancing the water solubility, biocompatibility, and stability of drugs, and reducing their toxicity [6]. In recent years, combination therapy utilizing nanocarriers for drug delivery has attracted significant attention for improving cancer treatment outcomes [7,8].

Sulforaphane (SFN) is a naturally occurring isothiocyanate found in cruciferous vegetables, notable for its cancer-preventive effects. Its mechanism involves multiple targeted effects, including the induction of phase II antioxidant enzymes, cell cycle arrest, and apoptosis [9,10,11]. According to studies, sulforaphane can enhance the efficacy of anticancer drugs while reducing their genotoxicity and cytotoxicity, such as bleomycin and doxorubicin. This dual mechanism may be associated with sulforaphane’s regulation of the cell’s antioxidant defense system and its modulation of the cell cycle and apoptosis [12,13,14]. Additionally, studies have reported that SFN can target cancer stem cells, which are believed to contribute to tumor progression, recurrence, and drug resistance [15]. All these studies suggest that sulforaphane may be a promising dietary anticancer therapeutic agent.

“Fighting fire with fire” is a common therapeutic principle in traditional Chinese medicine for treating malignant tumors [16]. Zhang Tingdong and colleagues achieved significant success in treating acute promyelocytic leukemia (APL) with arsenic trioxide (ATO) [17]. Cd is also a highly toxic metal, causing poisoning in various tissues of animals and humans, with a very long biological half-life and the liver and kidneys being the primary target organs in the human body [18,19,20]. Studies have shown that Cd induces mitochondria-dependent apoptosis associated with reactive oxygen species (ROS) in the liver and kidneys [21]. Metallothionein (MT), a non-specific protective protein, binds to most Cd in the body and reduces its adverse effects. However, compared to normal liver cells, MT expression is lower in liver cancer tissues [22]. Due to its toxic effects, some studies have found that cadmium (Cd) exhibits certain anti-tumor potential [23]. Cadmium-coordinated supramolecular complexes (TC 4ATS-Cd) demonstrate anti-proliferative effects on T-cell leukemia cells. Additionally, cadmium inhibits the growth of prostate cancer cells (PC-3) and exhibits cytotoxicity against epithelial-derived cancer cell lines [24]. Cd may also have some anti-cancer potential.

Methoxy poly (ethylene glycol)-poly (lactic-co-glycolic acid) (mPEG-PLGA) is an amphiphilic block copolymer and is commonly used as a nanocarrier [25,26]. Due to its higher stability, selective targeting, customizability, solubility, and low toxicity, it has attracted significant attention [27]. In addition, mPEG-PLGA nanoparticles can help reduce side effects by delivering specific drugs to target sites. Furthermore, the small size of the nanoparticles (10–100 nm) allows them to easily cross biological barriers in the system, thereby achieving selective accumulation and tumor destruction through the enhanced permeability and retention (EPR) effect [28,29,30].

This study introduces a nanodrug delivery system composed of cadmium (Cd)and sulforaphane (SFN) for the treatment of HepG2 cells. Through the coordination of γ-PGA with the carboxyl groups of Cd, nanoparticles are formed, thereby improving the solubility and encapsulation efficiency of Cd-γ-PGA [31]. The nanocarrier encapsulating both drugs is mPEG-PLGA, where the hydrophilic Cd-γ-PGA is encapsulated in the mPEG core, and the hydrophobic SFN is encapsulated in the PLGA hydrophobic shell. This allows for the simultaneous delivery of both hydrophilic and hydrophobic drugs to the same cancer cells in a predetermined ratio after injection. This innovative approach not only optimizes drug delivery but also allows for the reduction of drug dosage while achieving better therapeutic effects.

## 2. Materials and Methods

### 2.1. Materials and Reagents

Methoxy poly(ethylene glycol)-poly(lactide-co-glycolide) (mPEG–PLGA, molar ratio of D,L-lactic acid to glycolic acid, 75:25) was purchased from Jinan Daigang Biotechnology Co., Ltd. (Jinan, China). Sulforaphane was obtained from Yuanye Bio Co., Ltd. (Shanghai, China). Polyvinyl alcohol (PVA, 50 g, CAS:9002-89-5) was purchased from Solabio Technology Co., Ltd. (Beijing, China). Methylene chloride and cadmium chloride were obtained from Sinopharm Chemical Reagent Co., Ltd. (Beijing, China) and Shanghai Aladdin Bio-Chem Technology Co., Ltd. (Shanghai, China), respectively. Anti-beta-actin (Anti-ACTB) rabbit polyclonal antibody and HRP-conjugated goat anti-rabbit IgG were purchased from Southern Biotech (Birmingham, AL, USA). The enhanced cell counting kit-8 (CCK-8) assay kit, cell apoptosis detection kit, bicinchoninic acid (BCA) protein concentration assay kit, and radio-immunoprecipitation assay (RIPA) lysis buffer were obtained from Shanghai Beyotime Biotechnology Co., Ltd. (Shanghai, China). A minimum essential medium (MEM) with non-essential amino acids (NEAA) was purchased from Yuanye Bio Co., Ltd. (Shanghai, China).

### 2.2. Preparation of Cd-γ-PGA Conjugate

2 mL 20 mg/mL cadmium chloride solutions and 20 mg of γ-PGA were dissolved in 5 mL of deionized water. The mixture was stirred at 37 °C for 48 h and then extensively dialyzed with deionized water to remove free cadmium chloride. The conjugate mixture was analyzed using a Fourier Transform Infrared Spectrometer (FT-IR) to characterize its structure and confirm the characteristic absorption peaks of cadmium chloride and γ-PGA [31].

### 2.3. Preparation of NP-Cd-SFN

mPEG-PLGA (10 mg) was dissolved in 1 mL of dichloromethane, and 0.2 mL of Cd-γ-PGA solution (0.2 mg cadmium chloride) was added. The mixture was transferred to a centrifuge tube and subjected to ultrasonic homogenization for 3 min. Subsequently, 2 mL of 2% PVA solution was added to the mixture, which was then stirred at room temperature for 3 min. Thereafter, 0.2 mL of SFN dichloromethane solution was slowly added, and the mixture was homogenized again. The emulsion was gently poured into 10 mL of 0.6% PVA solution under stirring at room temperature for 10 min. After solvent evaporation under vacuum, the nanoparticles were collected by centrifugation at 13,000 rpm for 10 min at room temperature. The nanoparticles were washed twice with distilled water. Different drug concentration ratios were used to prepare nanoparticles of varying sizes for diverse drug delivery applications [32,33].

The encapsulation efficiency of Cd and sulforaphane was determined using inductively coupled plasma mass spectrometry (ICP-MS) and ultra-high-performance liquid chromatography-mass spectrometry (UHPLC-MS). The ICP-MS instrument parameters are as follows: radio frequency power of 1800 W, collision gas (helium) flow rate of 3.5 mL/min, plasma gas flow rate of 15 L/min, sample depth of 10.0 mm, carrier gas flow rate of 0.8 L/min, auxiliary gas flow rate of 0.8 L/min, helper gas flow rate of 0.8 L/min, nebulizer chamber type: cyclonic nebulizer chamber, nebulizer chamber temperature of 2 °C, and 3 measurement points per peak. The chromatographic column was C18 (250 × 4.6 mm, 5 μm) with a temperature set at 37 °C. The mobile phase consisted of (A) water and (B) acetonitrile, with a flow rate of 1.0 mL/min. The injection volume was 10 μL, and the quantification ion for SFN was set at 178.0 → 114.0.

Fluorescent dyes were employed as substitutes for Cd-γ-PGA to prepare NP-RhoB. After staining with 1% uranyl acetate, the nanoparticles were characterized using a transmission electron microscope (TEM, JEM-1400, JEOL, Tokyo, Japan). The particle size, polydispersity index (PDI), and zeta potential of the nanoparticles were measured using dynamic light scattering (DLS, Malvern Instruments Ltd., Malvern, UK).

### 2.4. Drug Release In Vitro

A total of 5 mg of NP-Cd-SFN was placed in 5 mL of phosphate-buffered saline (PBS) solution at pH 5.5 or pH 7.4 and transferred into a dialysis bag (molecular weight cutoff 1000 Da). The drug release experiment was conducted at 37 °C. At predetermined time intervals, 0.2 mL of the solution was collected for the quantitative determination of sulforaphane (SFN) and cadmium using ultra-performance liquid chromatography-mass spectrometry (UPLC-MS) and inductively coupled plasma mass spectrometry (ICP-MS), respectively. The instrument parameters for these analyses were consistent with those described in Section 2.3. After each collection, 0.2 mL of fresh PBS solution was added to maintain the overall volume.

### 2.5. Cytotoxicity Studies in HepG2 Cells

When HepG2 cells in the culture flask reach 80% confluence, they are digested with 0.25% trypsin. After digestion, the cells are centrifuged at 1300× *g* for 3 min to collect the cell pellet, which is then resuspended in a fresh complete culture medium. The cell suspension is counted to determine the cell density.

Cells are seeded into a 96-well culture plate at a density of 1 × 10^4^ cells per well. The plate is then incubated at 37 °C in a humidified atmosphere of 5% CO_2_ for 24 h. After incubation, the cells are observed under a microscope to ensure good adhesion and growth.

Different concentrations of SFN, Cd, SFN+Cd, NP-SFN, NP-Cd, and NP-SFN-Cd are added to the wells (100 μL per well). The relevant grouping information is shown in Table 1. The cells are incubated for another 24 h. Following this, the old medium is replaced with fresh medium (100 μL), and 10 μL of CCK-8 solution is added to each well. The plate is incubated for an additional 1 h, after which the optical density (OD) values are measured at 450 nm using a microplate reader.

### 2.6. Cellular Uptake

HepG2 cells were seeded in confocal dishes (Nunc, MA, USA). Following an overnight incubation period of 16–18 h, the cells were treated with free RhoB or NP-RhoB for 1 or 6 h. Subsequently, the cells were washed three times with phosphate-buffered saline (PBS) prior to imaging using a laser confocal fluorescence microscope (Nikon A1R HD25, Tokyo, Japan). The nucleus and lysosome were stained with Hoechst 3342 and LysoTracker Green DND 26 (Yuanye Bio Co., Ltd., Shanghai, China), respectively, under specified incubation conditions.

To quantitatively compare cellular uptake between free RhoB and RhoB NPs, HepG2 cells were seeded in 12-well plates. Upon reaching 70% confluence, the cells were treated with free RhoB or RhoB NPs for 6 h. After harvesting and washing three times with PBS, the cells were suspended in 0.2 mL of PBS and analyzed using an Accuri C6 flow cytometer (BD, NJ, USA).

### 2.7. Cell Apoptosis Study in HepG2 Cells

#### Incubation of RAW 264.7 Cells

HepG2 cells were seeded in 12-well plates at a density of 1 × 10^4^ cells/well. After an overnight incubation period, the cells were treated with SFN, Cd, SFN-Cd, NP-SFN, and NP-Cd-SFN for 24 h. Subsequently, the cells were harvested and co-stained with propidium iodide (PI) and Annexin V using the Annexin V-FITC apoptosis detection kit (Solarbio, Shanghai, China). Cell apoptosis was analyzed by flow cytometry.

### 2.8. Western Blot Assay

HepG2 cells were collected after different treatments by trypsinization and harvested. Total cellular protein was extracted using radio-immunoprecipitation assay (RIPA) lysis buffer (Beyotime, Shanghai, China). The concentration of protein was measured using a bicinchoninic acid (BCA) protein assay kit (Beyotime, China). A total of 50 µg of proteins was used for SDS-PAGE electrophoresis and transferred to 0.22 µm polyvinylidene fluoride (PVDF) membranes (Millipore, MA, USA) at 100 V for 60 min. Membranes were blocked with 5% non-fat milk in tris buffered saline with tween (TBST) for 1 h at room temperature. Primary antibodies targeting p53, NFR2, Bcl 2 (Abcam, Cambridge, UK) were diluted in blocking buffer and incubated with the membranes overnight at 4 °C. After washing three times with TBST, secondary antibodies (HRP-conjugated anti-rabbit IgG, Abcam, UK) were added and incubated for 1 h at room temperature. After washing three times, immunoreactivity was visualized using enhanced chemiluminescence (ECL) reagents (Beyotime, China) and imaged using a ChemiDoc XRS+ imaging system (Bio-Rad, CA, USA). ACTB was used as a loading control.

### 2.9. In Vitro Metallothionein Detection

HepG2 cells were collected and resuspended in fresh complete culture medium to prepare a cell suspension at a density of 5 × 10^4^ cells/mL. Cells were seeded into a 6-well cell culture plate (5 × 10^4^ cells/well) and incubated in a 5% CO_2_, 37 °C incubator for 24 h. After incubation, cells were treated with the corresponding drugs (SFN, Cd, SFN + Cd, NP-Cd-SFN) at concentrations of 10 µM. Cells were incubated for another 48 h, then the supernatant was collected by centrifuging at 3000× *g* for 10 min at 4 °C. The supernatant was stored at −80 °C for subsequent analysis. Metallothionein levels were detected using a Metallothionein ELISA kit (Abcam, UK) following the manufacturer’s instructions.

### 2.10. Statistics

All experiments were conducted in triplicate unless otherwise indicated. The results are presented as the mean ± standard deviation (SD). Statistical analysis was performed using a two-tailed Student’s *t*-test. A difference between the two groups was considered statistically significant when *p* < 0.05 (two-tailed).

## 3. Results and Discussion

### 3.1. Characterization of NP-Cd-SFN

**Figure 1:**
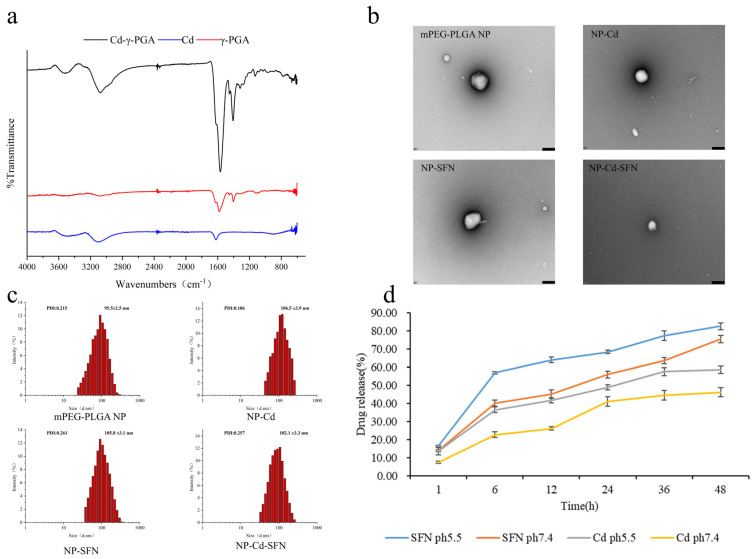
Characterization of NP-Cd-SFN. (**a**) Fourier transformed infrared (FT-IR) analysis of Cd, γ-PGA and Cd-γ-PGA Conjugate. (**b**) TEM image of mPEG-PLGA NP, NP-Cd, NP-SFN, and NP-Cd-SFN. Scale bar, 100 nm (**c**) Size distribution of mPEG-PLGA NP, NP-Cd, NP-SFN and NP-Cd-SFN. (**d**) The drug release curves of NP-Cd-SFN in pH 5.5 and 7.4 PBS buffer solutions over 48 h.

The water-soluble Cd-γ-PGA conjugate was successfully synthesized and confirmed via Fourier transform infrared (FT-IR), as shown in Figure 1a. The characteristic peak of Cd around 600 cm^−1^ corresponds to the ionic bond vibrations between Cl^−^ and Cd^2+^, primarily reflecting the ionic bond vibrations between Cl^−^ and Cd^2+^ [34]. The infrared spectrum of γ-polyglutamic acid (γ-PGA) mainly includes characteristic peaks from carboxyl groups (-COOH) and amide bonds (-NHCO-), with the C=O stretching vibration of carboxyl groups appearing in the range of 1650–1700 cm^−1^. The spectrum of Cd-γ-PGA exhibits similar characteristic peaks to Cd and γ-PGA, as they share the same functional groups. However, a new absorption peak was observed in Cd-γ-PGA around 1400 cm^−1^, corresponding to new coordination bonds formed between Cd and oxygen or nitrogen atoms in γ-PGA. Additionally, the spectrum of Cd-γ-PGA shows a characteristic peak at approximately 1620 cm^−1^, similar to Cd and γ-PGA at around 1600 cm^−1^, which is likely due to the displacement of C=O stretching vibrations caused by coordination effects. These characteristic peaks confirm the successful coupling of Cd with γ-PGA and the formation of new coordination bonds.

NP-Cd-SFN was prepared using an improved double emulsion method, with an encapsulation efficiency of 63.6 ± 2.8% for SFN and 86.5 ± 2.4% for Cd, as shown in Table 2. Subsequent experiments were prepared using the same method. The four types of nanoparticles exhibit spherical morphologies. Dynamic Light Scattering (DLS) results indicate that the blank mPEG-PLGA NP has a diameter of approximately 95.5 ± 2.5 nm, NP-Cd has a diameter of about 106.5 ± 3.9 nm, NP-SFN has a diameter of approximately 105.8 ± 3.1 nm, and NP-Cd-SFN has a diameter of about 102.1 ± 3.3 nm (Figure 1b,c). NP-Cd, NP-SFN, and NP-Cd-SFN all demonstrate slightly larger hydrodynamic diameters compared to the blank mPEG-PLGA NP, along with surface potentials of approximately −15 mV. These characteristics contribute to the nanoparticles’ prolonged circulation in the bloodstream and their ability to passively target tumors [35,36].

### 3.2. In Vitro Drug Release

The drug release behavior of NP-Cd-SFN was investigated in pH 5.5 and 7.4 PBS buffer solutions (Figure 1d). Both sulforaphane and cadmium (Cd) exhibited sustained release over 48 h, with the fastest release rate observed within the initial 6 h. At pH 5.5, the cumulative release efficiencies of SFN and Cd after 48 h were 82.53 ± 2.03% and 58.5 ± 2.07%, respectively. In contrast, at pH 7.4, the cumulative release efficiencies decreased to 75.58 ± 2.04% for SFN and 46.19 ± 2.56% for Cd. These results indicate that NP-Cd-SFN exhibits higher drug release efficiency in the mildly acidic environment (pH 5.5) compared to the neutral environment (pH 7.4). This suggests that NP-Cd-SFN may maintain stable drug release in the tumor microenvironment, potentially enhancing its anti-tumor effects and chemotherapeutic efficacy.

### 3.3. Stability and Safety of NP-Cd-SFN

The stability and colloidal properties of Cd-SFN-NP were evaluated by analyzing the variations in particle size and zeta potential over a 5-day period in 37 °C pH 7.4 PBS buffer and physiological saline. As shown in Figure 1, the particles maintained an average size of approximately 100 ± 2.6 nm, and the zeta potential remained relatively stable at approximately −14 ± 1.51 mV, exhibiting minimal variation over the 5-day period. These results demonstrate the excellent colloidal stability and physical integrity of the synthesized Cd-SFN-NP. Furthermore, this stability suggests that Cd-SFN-NP retains its structural integrity during in vivo metabolism, thereby preventing premature drug release and prolonging its half-life in the body. This enhanced stability is expected to improve the efficiency of tumor cell growth inhibition and enhance the chemotherapeutic effects of Cd-SFN-NP in vivo.

### 3.4. Cellular Uptake and Intracellular Localization

**Figure 2:**
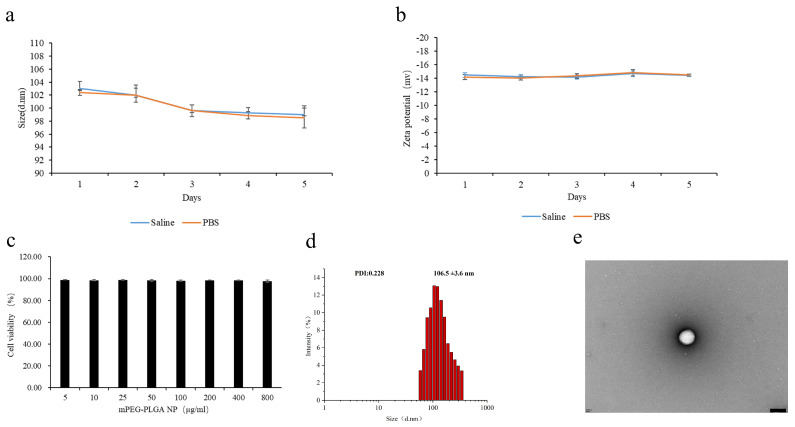
Stability and Safety of NP-Cd-SFN. (**a**) NP-Cd-SFN were dispersed in PBS buffer and Saline incubated at 37 °C for 5 days. Size of NP-Cd-SFN at different time points. (**b**) Zeta potential of NP-Cd-SFN at different time points. (**c**) Safety evaluation of nanocarriers: HepG2 cell viability after 24-h treatment with different concentrations of mPEG-PLGA NP. (**d**) Characterization of NP-RhoB. Particle size distribution graph of NP-RhoB. (**e**)TEM image of NP-RhoB.optimization. Scale bar, 100 nm.

To investigate the cellular uptake behavior of nanodrugs, NP-RhoB was prepared with physical and chemical properties similar to NP-Cd-SFN. NP-RhoB also exhibited a spherical morphology with a particle size of 106.5 ± 3.6 nm (Figure 2d,e). Free RhoB and NP-RhoB were incubated with HepG2 cells, followed by observation using a confocal laser scanning microscope.

**Figure 3:**
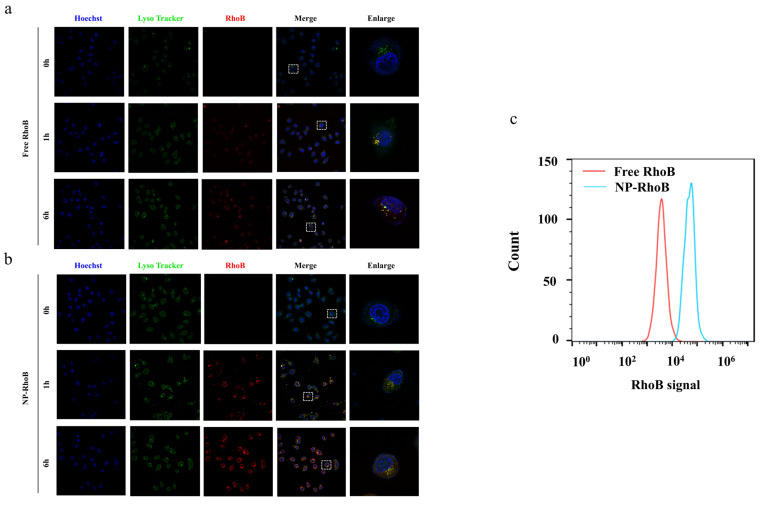
Cellular uptake and subcellular distribution of free RhoB and NP-RhoB. Cell nuclei were labeled with Hoechst 33342 (blue), and lysosomes were labeled with LysoTracker (green). (**a**) Confocal laser scanning microscopy images of HepG2 cells treated with free RhoB for different durations; (**b**) Confocal laser scanning microscopy images of HepG2 cells treated with NP-RhoB for different durations; (**c**) RhoB signals in HepG2 cells after 6-h incubation with free RhoB and NP-RhoB.

Free RhoB and NP-RhoB both exhibited time-dependent cellular uptake. After one hour of treatment, the majority of the red fluorescence from NP-RhoB in HepG2 cells overlapped with the green fluorescence of lysosomes, indicating that NP-RhoB was primarily internalized via endocytosis and entered lysosomes. After 6 h of treatment, the red fluorescence from NP-RhoB separated from the green fluorescence of lysosomes and was evenly distributed in the cytoplasm, suggesting that RhoB had translocated from lysosomes to the cytoplasm (Figure 3a,b). In the free RhoB group, the red and green fluorescence were uniformly distributed in the cytoplasm, indicating that free small molecules entered cells via passive diffusion. Compared to the free RhoB group, the NP-RhoB group exhibited stronger red fluorescence (RhoB signal) (Figure 3c), demonstrating that endocytosis of NPs is more efficient than passive diffusion of small molecules.

### 3.5. Evaluation of Cytotoxicity in HepG2 Cells

**Figure 4:**
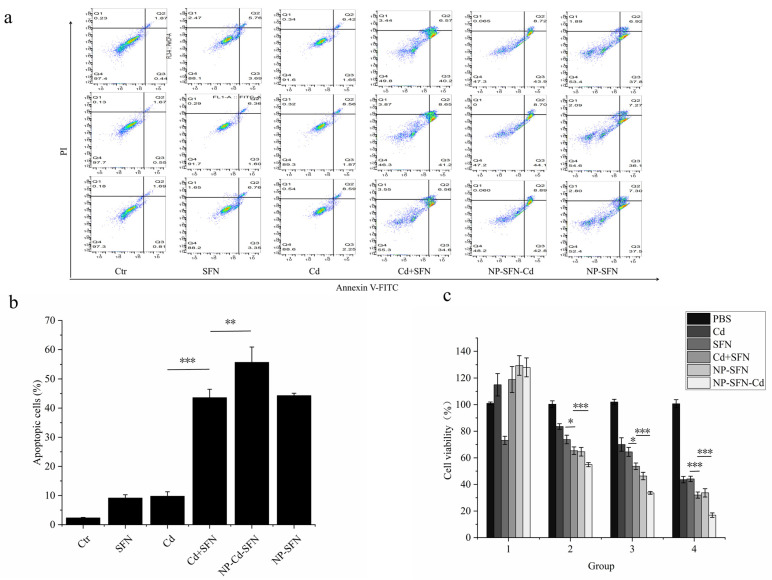
Evaluation of cytotoxicity. (**a**) The apoptotic effect of different drugs on HepG2 cells after 24-h treatment. (**b**) Evaluation of cytotoxicity in different nano-drug groups. (**c**) Quantitative analysis of apoptotic cells after 24-h treatment with different drugs. “*” denotes *p* < 0.05, and “***” denotes *p* < 0.001.

To evaluate the cytotoxic effects of each group of drugs on HepG2 cells, cytotoxicity assays, and apoptosis assays were conducted, with the results shown in Figure 4. The cytotoxic effects of all drug groups on HepG2 cells exhibited a dose-dependent manner. As shown in Groups 3 and 4, when SFN and Cd were administered individually, the cell viabilities were 70.03 ± 5.05% and 64.46 ± 3.33%, respectively, while for SFN + Cd combination treatment, the viabilities were 53.67 ± 2.45% and 32.00 ± 2.31%. These data demonstrated significant differences, indicating that the combination of SFN and Cd showed a significantly better effect than either drug alone. Additionally, the best cytotoxicity of NP-SFN was observed at 33.67 ± 3.11%, while that of NP-Cd-SFN reached 16.9 ± 1.68%. After encapsulation with the nanocarrier, NP-SFN and NP-Cd-SFN exhibited better effects compared to the free drugs at the same concentrations. These findings demonstrate that SFN and the nanocarrier enhance the cytotoxicity of Cd against HepG2 cells and improve its therapeutic efficacy.

**Figure 5:**
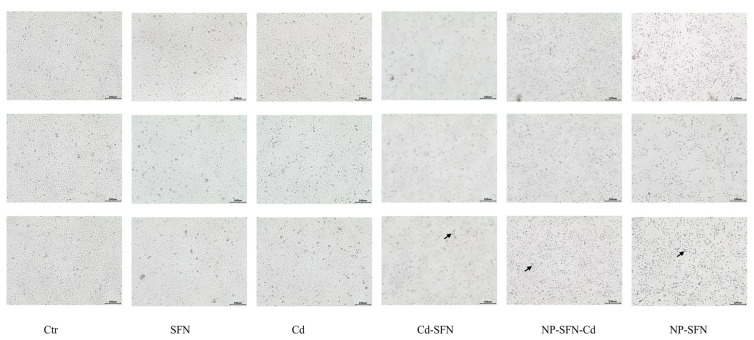
Morphological observation of apoptosis in HepG2 cells after 24-h treatment with different drugs. Scale bar, 100 μm.

After 24-h treatment with SFN, Cd, SFN + Cd, NP-SFN, and NP-SFN-Cd, HepG2 cells exhibited morphological changes, including cell shrinkage, indicative of an apoptotic trend, as shown in Figure 5. Among these treatments, the morphological changes were most pronounced in the SFN + Cd, NP-SFN, and NP-SFN-Cd groups. Flow cytometry analysis revealed that HepG2 cells underwent significant apoptosis after treatment, with early apoptosis, late apoptosis, and total apoptosis rates shown in Figure 4a. A bar chart analysis based on the total apoptosis rate is presented in Figure 4c, the NP-SFN-Cd group exhibited the highest apoptosis rate at 52.37 ± 5.18%, significantly higher than the SFN + Cd group (45.92 ± 2.87%), which in turn showed a significantly higher rate compared to the single drug treatment groups. Additionally, the NP-SFN group demonstrated an apoptosis rate of 44.3 ± 0.80%, significantly higher than the SFN single drug group. These results indicate that both the addition of SFN and the encapsulation of the nanocarrier enhance the efficacy of Cd.

### 3.6. Mechanistic Analysis of Apoptosis Induced by NP-Cd-SFN

**Figure 6:**
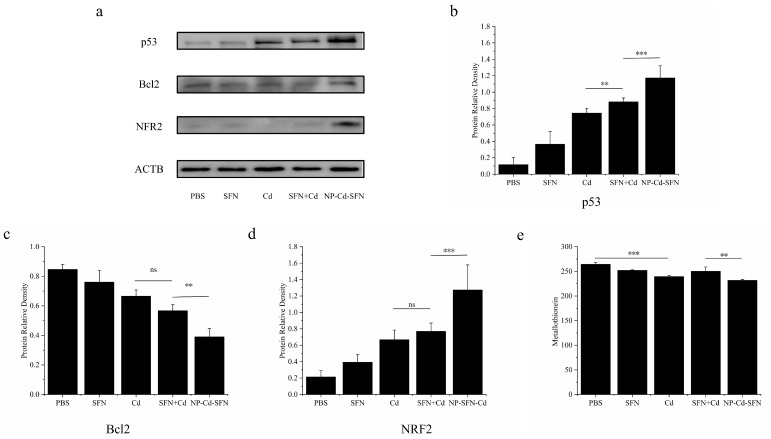
Mechanistic analysis of apoptosis induced by NP-Cd-SFN. (**a**) The effect of different drug treatments on the protein expression levels in HepG2 cells. (**b**) p53 protein expression level in HepG2 cells; (**c**) Bcl2 protein expression level in HepG2 cells; (**d**) NRF2 protein expression level in HepG2 cells. (**e**) Metallothionein content in HepG2 cells after different drug treatments. “**” denotes *p* < 0.005, and “***” denotes *p* < 0.001, ”ns” denotes no significant difference.

Western blot analysis (Figure 6a–d) demonstrated that compared to the Cd, SFN, and Cd + SFN groups, Bcl2 was significantly reduced and p53 was significantly increased in the NP-Cd-SFN group. These changes are closely associated with apoptosis. Additionally, Nrf2 was significantly elevated, indicating the activation of cellular defense mechanisms. The ultimate goal of anticancer therapy is to induce tumor cell apoptosis. Most chemotherapeutic drugs induce apoptosis by targeting anti-apoptotic proteins such as BcL2 and pro-apoptotic proteins like p53, which regulate cellular sensitivity at the mitochondrial level and determine cell fate [37,38]. The BCL2 protein family includes anti-apoptotic and pro-apoptotic molecules that primarily control cell sensitivity at the mitochondrial level, making it a key player in the response of cancer cells to various anticancer therapies. p53, a powerful tumor suppressor, inhibits tumor growth through multiple mechanisms [39,40]. The tumor suppressor protein p53 plays a critical role in the cellular response to DNA damage. Additionally, p53 suppresses inflammatory responses, and the loss of p53 function leads to excessive inflammation [41]. NRF2 is a regulator of cellular resistance to reactive oxygen species (ROS) and serves as a key transcription factor that regulates the expression of various cell-protective genes, such as GSH, GR, NQO1, and HO-1. It is closely associated with ROS-induced inflammatory responses [42]. The Keap1/Nrf2/ARE signaling pathway is a key therapeutic mechanism of sulforaphane. Nrf2 acts as a central regulator of cellular detoxification responses and redox state maintenance, which leads to the activation of cellular defense mechanisms and promotes detoxification processes [43].

Generally, cadmium exposure disrupts cellular signaling networks, leading to oxidative stress and inflammation, and induces apoptotic cell death [44]. Metallothioneins (MTs) are a class of metal-binding proteins primarily found in the liver, characterized by their low molecular weight and hydrophilic nature. They are rich in cysteine residues, and the presence of multiple cysteine residues with thiol (SH) groups endows MTs with a remarkable ability to bind metals and participate in redox reactions. This enables MTs to play a crucial role in regulating intracellular metal homeostasis and preventing associated toxicities [45]. Furthermore, MT was significantly lower in the NP-Cd-SFN group compared to the Cd + SFN group. Compared to the PBS group, Cd treatment led to a decrease in MT levels in the Cd group (Figure 6e), suggesting that Cd intervention depletes the limited MT in HepG2 cells, thereby exacerbating Cd’s toxic effects on these cells, primarily through mitochondrial oxidative stress and promoting cell apoptosis. Additionally, the addition of SFN derived from cruciferous vegetables significantly enhanced the inhibitory effect of Cd on HepG2 cells in the NP-Cd-SFN group, and the encapsulation of SFN within the nanocarrier further improved its efficacy.

## 4. Conclusions

SFN and Cd co-loaded mPEG-PLGA nanoparticles have been successfully prepared to enhance the therapeutic effects on HepG2 cells. The effective internalization of NP-Cd-SFN by HepG2 cells, due to their optimized particle size and zeta potential parameters, facilitates their uptake into the cells via endocytosis and subsequent entry into lysosomes. In the acidic microenvironment of cancer cells, this promotes the release of active drugs from the nanoparticle formulation, making it an ideal choice for targeted drug delivery. This increases the bioavailability of Cd and SFN in HepG2 cells and enhances the toxic effects of Cd on these cells, significantly improving the therapeutic efficacy of the drug. Overall, the addition of SFN and the encapsulation of mPEG-PLGA enhance the toxic effects of Cd on HepG2 cells.

## Figures and Tables

**Table 1:** Dosage of Different Groups.

Group	Cd (μg/mL)	SFN (μg/mL)
1	0.1	2
2	0.2	2
3	0.6	4
4	0.8	8

**Table 2:** Characterization and encapsulation efficiency of NP-Cd-SFN.

SFN (mg)	Cd (mg)	mPEG-PLGA (mg)	Cd Encapsulation Efficiency (%)	SFN Encapsulation Efficiency (%)	Size (nm)	Zeta Potential (mV)	PDI
0.25	0.2	10	86.5 ± 2.4%	61.2 ± 4.6	100.5 ± 3.8	−13.24 ± 1.8	0.203
0.5	0.2	10		63.6 ± 2.8	102.1 ± 3.3	−14.48 ± 1.4	0.257
1	0.2	10		55.3 ± 5.2	106.3 ± 4.3	−15.57 ± 2.6	0.175

## Data Availability

The original contributions presented in the study are included in the article, further inquiries can be directed to the corresponding author.
